# Weight Concerns and Use of Cigarettes and E-Cigarettes among Young Adults

**DOI:** 10.3390/ijerph15061084

**Published:** 2018-05-28

**Authors:** Brooke L. Bennett, Pallav Pokhrel

**Affiliations:** 1 Department of Psychology, University of Hawaii at Manoa, 2500 Campus Rd, Honolulu, HI 96822, USA; brooke37@hawaii.edu; 2 Cancer Prevention in Pacific Program, University of Hawaii Cancer Center, 701 Ilalo St, Honolulu, HI 96813, USA

**Keywords:** young adults, e-cigarettes, smoking, weight concerns

## Abstract

Higher weight concerns have been associated with higher cigarette smoking, particularly among women, partly because smoking is perceived to limit appetite for food. E-cigarettes are increasingly used as an alternative to combustible cigarettes and are widely believed to be less harmful than cigarettes. Currently it is not known whether weight concerns are associated with e-cigarette use among young adults. In this study, we tested the association between weight concerns and cigarette and e-cigarette use and use susceptibility among young adults. Cross-sectional data were collected from college students (N = 470; M age = 20.9; SD = 2.1; 65% women). Results indicated that weight concerns were significantly associated with lifetime and current cigarette smoking status, current cigarette smoking frequency and cigarette use susceptibility (among never smokers), adjusting for demographics variables. Weight concerns were not associated with lifetime or current e-cigarette use status or e-cigarette use susceptibility, adjusting for demographics and cigarette use status. However, higher weight concerns were associated with higher frequency of current e-cigarette use, adjusting for demographic variables and current cigarette smoking frequency. These findings imply that even though weight concerns may not motivate e-cigarette use as strongly as cigarette use, weight concerns may influence higher intensity of e-cigarette use among users.

## 1. Introduction

Since as early as the 1930s, tobacco marketing has directly or indirectly associated cigarette smoking with weight control [1]. Nicotine, one of the primary constituents of conventional cigarettes, is an appetite suppressant [2]. This has made smoking appealing for individuals who have high weight concerns, defined as elevated level of worry about current weight and fear of gaining weight [3]. Research demonstrates that on average, smokers tend to be thinner than non-smokers [4,5] and after smoking cessation, former smokers tend to gain 4–5 kg within the first year of abstinence [6].

Higher weight concerns have been found to be associated with higher current cigarette use among both adults [7] and adolescents [8]. Weight concerns appear to be associated with smoking initiation as well. Smoking cigarettes to control appetite and weight gain is prevalent among adolescents of both genders, although more so among girls than boys [7,8,9,10]. Howe et al. [11] found greater odds of smoking initiation linked with higher Body Mass Index (BMI) and high body dissatisfaction among both boys and girls. Similarly, dieting [12] and perceived importance of being thin [13] have been found to predict future smoking among girls.

In recent years, electronic- or e-cigarette use has steadily increased as an alternative to cigarette smoking [14]. In the United States, e-cigarettes are only loosely regulated and, therefore, are widely available for purchase and are generally easy to access [15]. Increased popularity of e-cigarettes may also be due to the widely accepted perception that e-cigarettes are healthier, safer or cleaner alternatives to conventional cigarettes [16]. Currently, relatively little is known about the relationship between weight concerns and e-cigarette use and susceptibility.

Given the known association between weight concerns and cigarette smoking, mainly among women, a positive association between weight concerns and e-cigarette use seems plausible because e-cigarettes are commonly used as cigarette substitutes and most users use e-cigarette for nicotine delivery [17]. A recent study showed that approximately 14% of current e-cigarette users who are trying to lose weight are actively using e-cigarettes to lose weight [18]. This study also found that among e-cigarette users, self-reported overweight status, relative to normal weight, was associated with the use of e-cigarette for weight loss. Another study [19] showed that e-cigarette users with current eating disorders had increased odds of reporting use of e-cigarettes for weight control, compared to e-cigarette users with no eating disorder history. To our knowledge, no study has examined weight concerns as a predictor of e-cigarette use and e-cigarette use susceptibility in a general sample of young adults, after adjusting for the effects of cigarette smoking status.

To address this gap, the present study aimed to test the relationship between weight concerns and conventional cigarette and e-cigarette use in a sample of young adult college students. First, based on past finding, it was hypothesized that higher weight concerns would be associated with greater likelihood of current cigarette use, experimentation, susceptibility, and higher current cigarette use frequency, after adjusting for demographic variables. Next, we tested the hypotheses that higher weight concerns would be associated with greater likelihood of current e-cigarette use and experimentation; higher current e-cigarette use frequency; and higher e-cigarette use susceptibility, after adjusting for cigarette smoking status and demographic variables (e.g., age, gender, and ethnicity). In addition, we tested gender differences in the associations between e-cigarette use, cigarette smoking, and weight concerns. This was achieved by testing the “gender X weight concerns” interaction effects on e-cigarette use and cigarette smoking.

## 2. Materials and Methods

### 2.1. Procedures

Participants were recruited in the fall of 2016 and spring of 2017 from two 4-year and four 2-year colleges belonging to a single university system and located on the island of Oahu in Hawaii, where 75% of Hawaii’s population resides. E-mail addresses of all 18–25-years-old students enrolled in the university system were obtained. From this pool of e-mail addresses, 2500 e-mail addresses were randomly selected in order to invite students to participate in the screener survey, with a goal of recruiting approximately 500 participants in the main study. The link to the screener survey was accompanied by an invitation text which described the study in generic terms, as a study on marketing and young adult health behavior. The screener survey asked questions about age, sex, tobacco, alcohol, and dietary behaviors. Invited students were given on average 2 weeks of time to respond and provided up to 3 reminders. Approximately 1300 students completed the screener survey, of which 742 were invited to participate in the main study. Those who were not invited included individuals who did not fall in the 18–25 years age range or never cigarette smokers or experimenters who responded after the quota for never smokers and experimenters was reached.

We intended to invite approximately equal numbers of current cigarette smokers, cigarette experimenters (i.e., those who had smoked less than 100 cigarettes in their lifetime and were current non-smokers), and never smokers to participate in the main study. However, fewer current cigarette smokers completed the screener survey than never smokers and experimenters. Hence, the first 298 and 296 never smokers and experimenters, respectively, who completed the screener were invited to participate in the study. All 148 current smokers who responded were invited to participate. Each individual was provided, on average, 2 weeks’ time and 3 reminders to complete the main study survey. Data collection was stopped after the targeted 500 participants responded. The total sample size for the current analysis was 470, after removing cases with more than 20% missing data.

### 2.2. Measures

Demographics. Age and gender were assessed with a single question each. Socioeconomic status was assessed in terms of parental/family income. Ethnicity was determined based on two items that were inclusive of ethnicities common in Hawaii (e.g., Japanese, Chinese, Korean) [20] The first ethnicity item provided participants with a list of racial/ethnic categories and asked them to “select all that apply” with regard to their ethnic/racial background. The second item was essentially the same as the first but asked participants to choose one racial/ethnic group that they identified with most.

Weight concerns. Weight concerns were assessed using the Stanford Weight Concerns Scale [3], which includes 5 items (α = 0.87). The items measure worry over weight and body shape (e.g., “How much more or less do you feel you worry about your weight and body shape than others of your gender and age?”), fear of weight gain (“How afraid are you of gaining 3 pounds?”), perceived fatness (“Do you feel fat?”), dieting history (“When was the last time you went on a diet”) and preoccupation with weight (“Compared to other things in life, how important is your weight to you?”). Score on each item was converted following a recommended method [3,21] to range between 0 and 100. A mean score across the 5 items was used for analysis.

Cigarette smoking. Data were collected on lifetime cigarette smoking (e.g., “How many cigarettes have you smoked in your entire life?” Response options: “I have never smoked a cigarette”, “1–100 cigarettes”, and “Over 100 cigarettes”), past-30-day cigarette smoking (“Within the last 30-days, on how many did you use cigarettes?” Response options: “0 days”, “1–2 days”, “3–5” days, …, “20–29 days”, “Used daily”), and current smoking behavior (“How do you describe your current cigarette smoking behavior?” Response options: “I don’t smoke”, “I smoke sometimes/occasionally”, “I smoke every day” [22]). Those who had never smoked a cigarette were classified as never smokers. Self-identified current smokers and/or past-30-day smokers were classified as current smokers.

E-cigarette use. Lifetime e-cigarette use was measured with a single question (“Have you ever used an electronic cigarette (e-cigarette) or a similar vaping device?” Response options: “Yes”, “No”). To assess current e-cigarette use, participants were asked: “How often, if at all, do you currently use an e-cigarette? (Response options: “Daily”, “Less than daily, but at least once a week”, “Less than weekly, but at least once a month”, “Less than monthly”, “Not at all”) [23]. 

Cigarette smoking susceptibility. Cigarette smoking susceptibility was assessed in terms of cigarette use intentions. Four items (α = 0.87) [24,25,26,27,28,29,30] were used to assess smoking intentions among never smokers (e.g., “Do you think that in the future you might experiment with cigarettes?”; “If one of your best friends were to offer you a cigarette, would you use it?”; response options included 1: “Definitely no” to 5: “Definitely yes”). For analysis, “definitely no” to all items resulted in the susceptibility variable being coded as 0. Any other response on any item resulted in the variable being coded as 1.

E-cigarette use susceptibility. E-cigarette use susceptibility was assessed among e-cigarette never users by using an e-cigarette use intentions scale which was adapted from the above-mentioned smoking intentions measure. Four items (α = 0.88) [24,25,26,27,28,29,30] were used (e.g., “Do you think that in the future you might experiment with e-cigarettes?”; “If one of your best friends were to offer you an e-cigarette, would you use it?”; response options included 1: “Definitely no” to 5: “Definitely yes”). For analysis, “definitely no” to all items resulted in the susceptibility variable being coded as 0. Any other response on any item resulted in the variable being coded as 1.

### 2.3. Data Analysis

Data were analyzed in SAS. Three types of multiple regression were run to test hypotheses. Hypotheses concerning weight concerns and e-cigarette and cigarette use susceptibility were tested using logistic regression. Hypotheses concerning weight concerns and cigarette and e-cigarette use status were tested using multinomial logistic regression. E-cigarette and cigarette use status variable each was coded to have three categories: never users, experimenters (those who had smoked/used but were not current smokers/users), and current users. Hypotheses concerning the associations between weight concerns and current cigarette and e-cigarette use frequencies were tested using negative binomial regression. Demographic (age, sex, parental income, and ethnicity) were entered as covariates in all models. All models testing the associations between weight concerns and e-cigarette use or susceptibility included cigarette smoking status as a covariate, in addition to the demographic variables. For all the above mentioned dependent variables we also tested, in separate models, gender as a moderator of the effects of weight concerns on cigarette/e-cigarette use following methods recommended for testing the effects of interactions between categorical and continuous variables [31].

## 3. Results

### 3.1. Participants

Table 1 shows participants’ characteristics in terms of demographics and cigarette and e-cigarette use prevalence. Participants were 18–25 years old, undergraduate college students. As is common among samples recruited from college campuses, the majority of the participants were women [32]. Participants represented the ethnic/racial diversity of Hawaii. A majority (53%) of the participants in the “Other” ethnic category were Native Hawaiian/Pacific Islanders, the rest represented African Americans (10%), Hispanics (23%), and other (14%). Of the never e-cigarette users (N = 197), 2.5% were current cigarette smokers, 19.8% were cigarette experimenters (i.e., those who had smoked less than 100 cigarettes in their lifetime and were current non-smokers), and 77.7% were never cigarette smokers. Of the e-cigarette experimenters (N = 155), 14.2% were current cigarette smokers, 58.1% were cigarette experimenters, and 27.7% were never cigarette smokers. Of the current e-cigarette users (N = 115), 46.9% were current cigarette smokers, 43.5% were cigarette experimenters, and 9.6% were cigarette never smokers.

### 3.2. Regression Analysis

As seen in Table 2, the current data indicated that weight concerns are statistically significantly associated with increased likelihood of having ever experimented with cigarettes, being a current cigarette smoker and, among never smokers, being more susceptible to smoking cigarettes in the near future, adjusting for demographic variables. For example, a unit increase in weight concerns was associated with 2% increase in the likelihood of being a current smoker. Further, among never smokers, a unit increase in weight concerns was associated with 3% increased likelihood of intentions to smoke cigarettes in the future. We did not find a statistically significant interaction effect for gender and weight concerns (*p* > 0.05).

As Table 3 shows, no significant association was found between weight concerns and being an e-cigarette experimenter, current e-cigarette user or being susceptible for e-cigarette use, after adjusting for demographic variables and cigarette smoking variables. In addition, we did not find a significant gender X weight concerns interaction effect.

Table 4 shows the results of the negative binomial regression analyses examining the associations between weight concerns and current cigarette smoking and e-cigarette use frequencies. Higher weight concerns were associated with higher frequency of current cigarette smoking as well as e-cigarette use. A unit increase in weight concerns was associated with 1.01 (i.e., exponential of 0.02) times, or 1%, increase in the frequency of current e-cigarette, holding all covariates constant. We did not find any significant gender X weight concerns interaction effect. 

## 4. Discussion

This study examined the relationships between weight concerns and e-cigarette use and conventional cigarette smoking in a population sample of young adults. As expected, higher weight concerns were found to be significantly associated with higher likelihood of lifetime and current cigarette smoking status, current cigarette smoking frequencies and higher cigarette smoking susceptibility among cigarette never smokers, adjusting for demographic variables. We did not find support for the hypothesis that there is a significant association between weight concerns and e-cigarette use status or e-cigarette use susceptibility after adjusting for cigarette smoking and demographic variables. However, we found that higher weight concerns were associated with higher current e-cigarette use frequency. Lastly, the current data did not indicate that the association between weight concerns and cigarette smoking or e-cigarette use is moderated by gender. To our knowledge, this is the first study to have examined the association between weight concerns and e-cigarette smoking in a general sample of young adults taking into consideration the potential confounding effects of cigarette smoking.

Our finding that weight concerns accounted for a significant portion of the variance for both lifetime and current conventional cigarette use is consistent with the majority of existing literature which suggests that weight concerns can aid continued cigarette smoking and discourage smoking cessation [7]. Additionally, the present study showed that weight concerns may explain a significant portion of the variance in smoking susceptibility for non-smokers. This finding is consistent with the existing literature which links higher weight concerns with smoking initiation [7,11].

Our findings related to weight concerns and e-cigarette use and e-cigarette use susceptibility may be partially explained by existing literature on perceptions of e-cigarettes. Research comparing perceptions of conventional and electronic cigarettes among current smokers has found that conventional cigarettes are perceived as being better for weight control [16]. E-cigarettes tend to deliver reduced amounts of nicotine compared with combustible cigarettes [33]; hence, it is likely that weight concerns may not motivate e-cigarette use as strongly as cigarette smoking among young adults. However, for those who use e-cigarette, higher weight concerns is likely to be associated with increased frequency of e-cigarette use, presumably because of the need for higher levels of nicotine. Our findings related to weight concerns, smoking, and gender may be partially explained by our mixed-gender sample. The majority of past research which has examined weight concerns and smoking, has used an exclusively female sample. It is possible that individuals with higher weight concerns are more likely to smoke conventional cigarettes, regardless of gender.

The current study is different from the previous two studies that examined e-cigarette use for weight control [18,19] in at least two important ways. To begin with, our dependent variables of lifetime and current e-cigarette use and e-cigarette use susceptibility did not refer to use for weight control. We were interested in examining the association between higher weight concerns and e-cigarette use at the population level. Secondly, the previous studies were focused on current e-cigarette users who were trying to lose or control weight [18] or showed eating disorder symptomatology [19]. Thus, the two studies examined correlates of use of e-cigarettes specifically for weight control in samples of current e-cigarette users who were struggling with weight or eating disorders. In other words, the studies examined factors that set apart individuals with weight concerns who used e-cigarettes for weight control versus individuals with weight concerns who used e-cigarettes for other reasons. Given their focus on current e-cigarette users, our study may be comparable to these studies in regard to the relationship between weight concerns and current e-cigarette use frequency. In this sense, our finding that higher weight concerns is associated with increased current e-cigarette use frequency is consistent with the previous studies’ findings that among e-cigarette users with weight concerns or eating disorders, those with higher weight concerns or more serious eating disorder symptomatology are significantly more likely to use e-cigarettes.

A significance of the present study is that it contributes to the limited research on the relationship between weight concerns and e-cigarette use. Our results suggest that current perceptions of e-cigarette use as a less intense form of smoking [33] may be limiting their use for weight control and that weight control motives may not influence young adult to initiate e-cigarette use. Results of the present study may also have implications for tobacco control efforts. In current programs targeting conventional cigarettes, there is usually an emphasis on the use of cigarettes for weight control [34]. However, currently there is no clear understanding as to how potential use of e-cigarettes for weight control should be approached. With improvements in nicotine delivery efficiency of e-cigarette products, the use of e-cigarettes for weight control motives may increase. Our finding already shows that among current users, higher weight concerns may be associated with higher e-cigarette use frequencies. Hence, from a harm reduction point of view, it is possible that more smokers who smoke for weight control may be encouraged to switch over to using e-cigarettes. However, from the point of view of youth e-cigarette use initiation, even though the current findings suggest that e-cigarette use status is not associated with weight concerns, prevention scientists need to be vigilant about the prospect of increased e-cigarette use among youth for weight control reasons.

There are limitations to the present study that need to be considered. First, the present research used a sample of young adults between the ages of 18–25. Although this age range was a focus because of the high prevalence of e-cigarette use [14], there is a possibility that our findings may not generalize to older or younger populations. Secondly, the current data were cross-sectional; hence, no causal inferences may be made based on our findings. Thirdly, because the dual user subsample was too small in the current study, we were not able to examine the associations between e-cigarette use and weight concerns separately among dual users.

Further longitudinal research is needed to examine the relationship between appetite, weight control, weight concern, and e-cigarettes. In addition, the relationships in the present study may need to be examined in samples from other age groups, including adolescents and older adults. Adolescents at greatest risk for initiation of conventional cigarette smoking [35] and older adults are more likely to quit smoking [36].

## 5. Conclusions

In conclusion, the present study demonstrated that while weight concerns remain a relevant predictor of lifetime cigarette smoking, current cigarette smoking, and cigarette smoking susceptibility, weight concerns may not be a robust predictor of e-cigarette use status. However, higher weight concerns may be associated with frequency of current e-cigarette use.

## Figures and Tables

**Table 1:** Participant characteristics (N = 470).

Variables		Mean (SD)	Frequency
Age		20.9 (2.1)	
Gender			
	Men		34.8%
	Women		65.2%
Ethnicity			
	White		27.5%
	Asian		38.4%
	Filipino		16.0%
	Other		18.1%
Parental income			
	$0–$39,999		21.2%
	$40 K–$59,999		14.4%
	$60 K–$79,999		16.2%
	$80 K–$99,999		14.4%
	$100 K–$119,999		13.5%
	$120 K and over		20.4%
Cigarette smoking status			
	Never smoker		43.7%
	Experimenter		38.5%
	Current smoker		17.8%
E-cigarette use status			
	Never user		42.5%
	Experimenter		33.0%
	Current user		24.5%
Any cigarette use intention			10%
Any e-cigarette use intention			31%
Weight concerns ^1^		41.0 (SD = 25.6)	

Note: SD = Standard deviation. ^1^ Range = 0–100, Median = 38.1, 25% Quartile = 20.0, 75% Quartile = 60.0.

**Table 2:** Associations of weight concerns with cigarette smoking status and cigarette smoking susceptibility.

	Cigarette Experimentation		Current Cigarette Use		Cigarette Use Susceptibility ^1^ (N = 207)	
	(N = 470)					
Independent Variable	Odds Ratio (95% Confidence Interval)					
	Unadjusted	Adjusted	Unadjusted	Adjusted	Unadjusted	Adjusted
Weight Concerns	1.01 (1.001, 1.02) **	1.01 (1.001, 1.02) *	1.02 (1.004, 1.03) **	1.02 (1.01, 1.03) **	1.02 (1.004, 1.04) *	1.03 (1.007, 1.05) **

Note: ^1^ Susceptibility was examined among never cigarette smokers only. The adjusted model included age, sex, ethnicity, and parental income as covariates. * *p* < 0.05, ** *p* < 0.01, *** *p* < 0.001.

**Table 3:** Associations of weight concerns with e-cigarette use status and e-cigarette use susceptibility.

Independent Variable	All Sample (N = 470)				Never Cigarette Smokers (N = 207)				Never E-Cigarette Users (N = 197)		Never Smokers and Never E-Cigarette Users (N = 153)	
	E-Cigarette Use Experimentation		Current E-Cigarette Use		E-Cigarette Use Experimentation		Current E-Cigarette Use		E-Cigarette Use Susceptibility		E-Cigarette Use Susceptibility	
	Odds Ratio (95% Confidence Interval)											
	Unadjusted	Adjusted	Unadjusted	Adjusted	Unadjusted	Adjusted	Unadjusted	Adjusted	Unadjusted	Adjusted	Unadjusted	Adjusted
Weight concerns	1.01 (1.004, 1.02) **	1.008 (0.99, 1.02)	1.01 (1.001, 1.02) *	1.004 (0.99, 1.02)	1.02 (1.001, 1.03) *	1.01 (0.99, 1.02)	1.006 (0.98, 1.03)	1.002 (0.97, 1.03)	1.004 (0.99, 1.02)	1.004 (0.99, 1.02)	1.005 (0.99, 1.02)	1.006 (0.99, 1.02)

Note: Adjusted models included age, sex, ethnicity, parental education, and cigarette smoking status (where relevant). * *p* < 0.05, ** *p* < 0.01, *** *p* < 0.001.

**Table 4:** Associations of weight concerns with current cigarette and e-cigarette use frequencies.

Independent Variable	Cigarette Use (N = 470)		E-Cigarette Use (N = 470)	
	B (SE) ^1^			
	Unadjusted	Adjusted	Unadjusted	Adjusted
Weight concerns	0.02 (0.005) *	0.02 (0.006) *	0.01 (0.003) *	0.01 (0.004) *

Note: ^1^ B = Negative binomial regression coefficient, SE = standard error. * *p* < 0.05, ** *p* < 0.01, *** *p* < 0.001. Adjusted models included age, sex, ethnicity, parental income, and cigarette smoking status (in the case of e-cig).
